# *Cutibacterium acnes* Infection Induces Type I Interferon Synthesis Through the cGAS-STING Pathway

**DOI:** 10.3389/fimmu.2020.571334

**Published:** 2020-10-15

**Authors:** Katrin Fischer, Roland Tschismarov, Andreas Pilz, Susy Straubinger, Sebastian Carotta, Andrew McDowell, Thomas Decker

**Affiliations:** ^1^Max Perutz Labs, Department of Microbiology, Immunobiology and Genetics, University of Vienna, Vienna Biocenter, Vienna, Austria; ^2^Themis Bioscience GmbH, Vienna, Austria; ^3^Department of Cancer Research, Boehringer Ingelheim RCV GmbH & Co KG, Vienna, Austria; ^4^Nutrition Innovation Centre for Food and Health (NICHE), School of Biomedical Sciences, Ulster University, Coleraine, United Kingdom

**Keywords:** *Cutibacterium acnes*, interferon, *Listeria* (L.) *monocytogenes*, cyclic GMP-AMP synthase, stimulator of interferon genes, TIR-domain-containing adapter-inducing interferon-β, NFkB = nuclear factor kappa b, STAT (signal transducer and activator of transcription)

## Abstract

*Cutibacterium* (previously *Propionibacterium*) *acnes* is an anaerobic, Gram-positive commensal of the human body. The bacterium has been associated with a variety of diseases, including acne vulgaris, prosthetic joint infections, prostate cancer, and sarcoidosis. The accumulation of *C. acnes* in diseases such as acne and prostate cancer has been shown to correlate with enhanced inflammation. While the *C. acnes*-induced proinflammatory axis, *via* NF-κB and MAPK signaling and inflammasome activation, has been investigated over the last few decades, the potential role of *C. acnes* in triggering the type I interferon (IFN-I) pathway has not been addressed. Our results show that *C. acnes* induces the IFN-I signaling axis in human macrophages by triggering the cGAS-STING pathway. In addition, IFN-I signaling induced by *C. acnes* strongly depends on the adapter protein TRIF in a non-canonical manner; these signaling events occurred in the absence of any detectable intracellular replication of the bacterium. Collectively, our results provide important insight into *C. acnes*-induced intracellular signaling cascades in human macrophages and suggest IFN-I as a factor in the etiology of *C. acnes*-induced diseases. This knowledge may be valuable for developing novel therapies targeting *C. acnes* in diseases where the accumulation of the bacterium leads to an inflammatory pathology.

## Introduction

*Cutibacterium acnes* (previously *Propionibacterium acnes*) is an anaerobic, Gram-positive bacterium that preferentially colonizes the human skin, as well as mucosal surfaces (1, 2). While *C. acnes* has traditionally been considered a non-pathogenic resident of the human microbiota, current literature challenges this notion. In particular, *C. acnes* has been shown to play a morbific role during the inflammatory skin disease acne vulgaris (3), as well as prostate disease (2, 4, 5) and infections following implant surgeries (6–10). The bacterium produces a range of pathogenic factors, including proteases, lipases and chemotactic factors for macrophages, neutrophils and lymphocytes (11), and promotes innate immune cells such as macrophages to secrete inflammatory cytokines, particularly tumor necrosis factor α (TNF-α), interleukin (IL)-6, IL-8, IL-12, and IL-1 family members (12–15). Deciphering how *C. acnes* triggers the innate immune response can be helpful for understanding diseases where the accumulation of *C. acnes* plays a role, as well as for the development of host-directed novel therapies.

Type I Interferons (IFN-I) contribute to many aspects of immunity with a main focus on anti-viral responses (16, 17). IFN-I contribute to inflammatory responses by stimulating and amplifying the secretion of chemokines and other immune mediators (18). Activation of the IFN system in the defense of bacterial pathogens is not always beneficial, and may have detrimental effects on the host during infection with certain pathogens (18). For instance, enhanced IFN-I production during *Listeria monocytogenes* (*L.m.*) infections is associated with decreased innate immunity and reduced host resistance (19). In general, there are several possibilities for pathogens to stimulate the interferon pathway in host cells. For example, conserved structures of bacterial cell surfaces can bind and activate membrane-bound Toll-like receptors (TLR), with TLR4 as the main receptor for IFN-I induction. IFN stimulation *via* the TLR4 axis requires the adapter molecule TIR-domain-containing adapter-inducing interferon-β (TRIF) (20, 21). As TLR4 recognizes lipopolysaccharides (LPS) derived from Gram-negative bacteria, direct recognition of *C. acnes* by TLR4-TRIF appears unlikely. However, in some macrophage populations, such as those residing in the mouse peritoneum, TRIF signaling connects to TLR2, thus coupling the recognition of Gram-positive bacteria at the cell surface, or in endosomes, to IFN-I synthesis (22). On the other hand, host cells can react to cytosolic pathogenic intrusions by recognizing either foreign RNA (RIG-I-like receptors; RLR), intracellular bacterial cell surface components (NOD-like-receptors; NLR) or cytoplasmic DNA (cytosolic DNA-binding sensors). Cyclic GMP-AMP synthase (cGAS) is arguably the most prominent DNA sensing receptor found in the cytoplasm with relevance during bacterial infections (23, 24). DNA recognition by the sensor protein cGAS causes the enzyme to produce cyclic GMP-AMP (cGAMP), a cyclic di-nucleotide that associates with and activates stimulator of interferon genes (STING), an essential scaffold for the activation of interferon regulatory factors (IRF). As essential components of active IFN-I gene promoters, IRFs play a critical role in the control of IFN-I production (25). Some intracellular bacteria, most prominently *L.m.*, secrete cyclic-di-nucleotides that further stimulate the adapter STING in a cGAS-independent manner (26, 27). The potential role of *C. acnes* in triggering the interferon signaling axis, and how it compares in this regard to intracellular *L.m.*, has not been considered yet and will be the main focus of this study.

With our results we provide evidence that the Gram-positive bacterium *C. acne*s triggers the interferon signaling axis in human macrophages. In accordance with published data (12, 21, 28, 29) we demonstrate a strong inflammatory cytokine response, as well as the activation of the inflammasome. We further show that infection with *C. acnes* stimulates IFN-I production *via* the cGAS-STING pathway with a strong dependency on the adapter molecule TRIF. In contrast to *L.m.*, *C. acnes*-induced interferon production shows complete dependence upon the cytoplasmic DNA sensor cGAS, suggesting DNA as the only trigger. While the pro-inflammatory cytokine response shows little variation between *C. acnes* strains, not all strains elicit a strong interferon response. However, for the tested strains the mechanism behind the induction of interferon signaling in *C. acnes* infected macrophages, is the TRIF-dependent activation of the cGAS-STING pathway.

## Materials and Methods

### Cell Culture and Differentiation

Human monocytic THP-1 cells (ATCC #TIB-202 and Invivogen #thp-isg) and U-937 cells (ATCC #CRL-1593.2) were maintained in RPMI 1640 culture medium (Sigma-Aldrich) supplemented with 10% (v/v) heat-inactivated fetal bovine serum (FBS) (Sigma-Aldrich) and 1% Penicillin/Streptomycin (both Sigma-Aldrich) (referred to as “complete medium”).

For the differentiation into macrophage-like cells, human monocytic THP-1 or U-937 cells were seeded in complete medium supplemented with 100nM phorbol 12-myristate 13-acetate (PMA) (Sigma-Aldrich #P1585). Cells were differentiated for 2 days at 37°C and 5% CO_2_ atmosphere. On the 3rd day, media was exchanged to RPMI 1640 supplemented with 10% (v/v) FBS and incubation at 37°C and 5% CO_2_ was continued for an additional 24 h.

HaCaT cells obtained from the German Cancer Research Center (DKFZ, Heidelberg, Germany) were maintained in DMEM culture medium (Sigma-Aldrich) plus 10% (v/v) FBS and 1% Pen/Strep and kept at 37°C in a 5% CO_2_ atmosphere. Cells were split twice a week by washing once with PBS, incubating for 10 min with 0.05% EDTA in PBS at 37°C followed by a 10 min incubation with Trypsin-EDTA at 37°C.

### Isolation of PBMCs and Monocytes From Human Blood

Peripheral blood mononuclear cells (PBMC) were isolated by density gradient centrifugation with Lymphoprep™ (StemCell Technologies, #07851) from fresh buffy coats of three healthy donors received from the Austrian Red Cross. Erythrocytes were removed using ACK lysing buffer (Gibco, #A10492-01). After three washing steps, cell viability was measured (viability above 95%). Next, monocytes were isolated from PBMCs by negative selection using EasySep™ Human Monocyte Isolation Kit (StemCell Technologies, #19359) showing a viability above 91%. Monocytes were seeded on the same day in RPMI 1640 plus 10% (v/v) FBS and infected the following day.

### Differentiation of Primary Monocytes Into Monocyte-Derived Macrophages

Primary monocytes were seeded on the day of isolation in complete medium in 6-well plates. For differentiation, cells were stimulated with 100 ng/ml of recombinant human M-CSF (a kind gift from L. Ziegler-Heitbrock, Helmholtz Center, Munich, Germany). At day 4, media were refreshed (complete medium plus M-CSF). On day 6, media were exchanged to medium containing only 10% (v/v) FBS. Infection experiments were performed with primary monocyte-derived macrophages at day 7.

### Bacterial Preparation and Infection

All *C. acnes* strains were cultured in brain-heart-infusion (BHI) media (BD Bioscience) at 37°C under anaerobic conditions using BD GasPak™ (BD Bioscience). Bacteria were collected at exponential growth phase (around 24 h after inoculation), washed twice with 1x phosphate buffered saline (PBS) and resuspended in RPMI 1640 supplemented with FBS. All centrifugation steps were performed at 10,000x g for 5 min. The following strains were used: NCTC737 (phylotype IA_1_; ATCC6919); P. acn31 (phylotype IA_2_); KPA171202 (phylotype IB; DSM16379); PV66 (phylotype IC); ATCC11828 (phylotype II); Asn12 (phylotype III) (see **Table S1**). Infection was performed with a multiplicity of infection (MOI) of approximately 50. At different time-points, cells were washed twice with 1x PBS and lysed for either RNA or protein isolation.

*L.m.* strain LO28 and the LLO-deficient strain LO28Δ*hly* were grown overnight in BHI media at 37°C with continuously shaking. After reaching stationary phase, bacteria were washed twice with 1x PBS and resuspended in RPMI 1640 supplemented with 10% (v/v) FBS. Centrifugation was performed at 10,000x g for 5 min. Cells were infected with a MOI of approximately 40 for 1 h at 37°C. Media was exchanged to RPMI 1640 supplemented with 10% (v/v) FBS and 50 µg/ml gentamycin (MP Biomedicals, Santa Ana, US). After 1 more h, media was replaced with RPMI 1640 supplemented with 10% (v/v) FBS and 10 µg/ml gentamycin. After different time-points, cells were washed twice with 1x PBS and lysed for RNA or protein isolation.

LPS (Sigma-Aldrich #L2637) was used at a final concentration of 0.4 µg/ml.

### Colony-Forming-Unit Assay

THP-1 and U-937 cells were seeded in 96-well plates and differentiated using PMA (see above). Cells were infected with *C. acnes* strain NCTC737, P. acn31, KPA171202, PV66, ATCC11828 and Asn12 or *L.m.* strain LO28 or *L.m.* mutant strain LO28Δ*hly*. After 1-h post infection, media of all wells was exchanged to RPMI 1640 supplemented with 10% (v/v) FBS and 50 µg/ml gentamycin. After another hour, media was replaced by RPMI 1640 supplemented with 10% (v/v) FBS and 10 µg/ml gentamycin. After each timepoint, cells were washed twice with 1x PBS. Subsequently, cells were burst open by adding sterile nuclease-free water. Dilution series was made, and bacteria plated on either Brucella blood agar (*C. acnes*; bioMérieux Austria GmbH) or BHI plates (*L.m.*). Brucella blood agar plates were incubated for 3 days at 37°C under anaerobic conditions. BHI plates with *L.m.* were kept for 1.5 day at 37°C.

### RNA Isolation, cDNA Synthesis, and RT-qPCR

Total RNA isolation was performed using the NucleoSpin RNA II kit (Macherey-Nagel, Catalog #740955). For the synthesis of cDNA, Oligo (dT18) primer and RevertAid Reverse Transcriptase (both Thermo-Fisher Scientific) were used. Real-time quantitative-PCR was run on Eppendorf Mastercycler using SybrGreen (Promega Catalog # A6002). Primers for real-time qPCR are listed in **Table S2**.

### Western Blot

THP-1 cells were lysed in Laemmli buffer (120 mM Tris-HCl, pH 8, 2% SDS, and 10% glycerol) and protein concentrations measured using Pierce™ BCA Protein Assay kit (Thermo-Fisher Scientific). A total of 20 µg of protein were resuspended with SDS-loading dye (50% β-Mercaptoethanol, 0.02% Bromphenolblue), boiled and loaded on a 10% SDS polyacrylamide gel. Proteins were transferred on a nitrocellulose or PVDF membrane for 16 h at 200 mA and 2 h at 400 mA at 4°C using a carbonate transfer buffer (3 mM Na_2_CO_3_, 10mM NaHCO_3_, and 20% ethanol). Afterwards, membranes were blocked in 5% (w/v) milk powder in TBS-T for 1–2 h at room temperature. Membranes were incubated with the appropriate primary antibody overnight at 4°C while shaking: α-Tubulin (Sigma Aldrich, Catalog # T9026, 1:5000); GAPDH (Millipore, Catalog # ABS16, 1:3000); STAT1 (Santa Cruz, Catalog # SC346, 1:1000); Phospho-STAT1 (Tyr701; Cell signaling, Catalog # 9167, 1:1000); IRF9 (Santa Cruz, Catalog # sc10793, 1:1000); IκB-α (Cell signaling, Catalog #9242, 1:1000); MyD88 (Cell signaling, Catalog # 4283, 1:1000); TRIF (Cell signaling, Catalog # 4596, 1:1000); STING (Cell signaling, Catalog # 13647, 1:1000); cGAS (Cell signaling, Catalog # 15102, 1:1000). The membrane was then washed three times with TBS-T before incubating for 1–2 h at room temperature with the appropriate secondary antibody (Jackson ImmunoResearch Inc., Catalog # 111-035-003, 1:6000, and Catalog # 115-035-144, 1:6000). After three washing steps with 1x TBS-T, the membrane was incubated with SuperSignal West Pico Chemiluminescent substrate (Thermo-Fisher Scientific) and developed using the ChemiDoc™ Imaging system from Bio-Rad.

### Luminex Assay

ProcartaPlex Mix&Match human 8-plex from Affymetrix eBioscience was used for measuring cytokine levels in the supernatant of *C. acnes* infected and uninfected cells. Protein levels were measured at Affymetrix eBioscience, Campus Vienna Biocenter 2, 1030 Vienna. The kit was used according to the manufacturer’s specifications.

### THP1 ISG Reporter Activity

Wildtype THP1-Blue™ ISG cells were differentiated in a 96-well plate (5 x 10^4^ cells/well) with PMA as described above. Cells were infected with either *L.m.* strain LO28 or *C. acnes* strains NCTC737 and Asn12 or stimulated with 0.4µg/ml LPS for either 24, 48, or 72 h. After each time-point, 20 µl of the supernatant was added to 180µl of QUANTI-Blue™ solution (Invivogen, Cat. # rep-qbs) in a flat-bottom 96-well plate and incubated at 37°C. Absorbance at 620 nm was measured using BioTek Synergy H1 Plate reader.

### Genome-Editing *via* CRISPR-Cas9 System

The guide RNAs of human *MYD88*, *TICAM1* (gene encoding human TRIF) and *IRF9* were designed using Broad Institute GPP Web Portal (https://portals.broadinstitute.org/gpp/public/analysis-tools/sgrnadesign). *LacZ* gRNA (non-targeting control) (tgcgaatacgcccacgcgat), *MYD88* gRNA (TGTCTCTGTTCTTGAACGTG); *TICAM1* gRNA (GGAGAACCATGGCATGCAGG); *IRF9* gRNA (ATACAGCTAAGACCATGTTC; published in Platanitis et al. (30)). Oligos were ligated into the LentiCRISPRv2 plasmid. LentiCRISPRv2 was a gift from Feng Zhang (Add gene plasmid # 52961; http://n2t.net/addgene:52961; RRID : Addgene_52961) (31). THP-1 cells were transduced, and single cells selected.

Vector for the guide RNAs of human *TMEM173* (gene encoding human STING) and *CGAS* were ordered from Sigma-Aldrich (U6gRNA-Cas9-2A-GFP-TMEM173 and U6gRNA-Cas9-2A-GFP-MB21D1). *TMEM173* gRNA (GGGCCGACCGCATTTGGGAGGG); *CGAS* gRNA (CGTCGGGCTGCTGAACACCGGG).

### Statistical Information

Data derived from RT-qPCR represent the mean values in a min/max box plot. Differences in mRNA expression were compared using the two-tailed unpaired t-test of the log transformed values. Differences in bacterial loads (CFU) were compared using the two-tailed unpaired t-test. All statistical analysis was performed using GraphPad Prism. P-values: ns P > 0.05; *P ≤ 0.05; **P ≤ 0.01; ***P ≤ 0.001. Number of biological replicates is stated in the corresponding figure legend.

## Results

### *C. acnes* Intracellular Degradation in Human Macrophages

**Figure 1:**
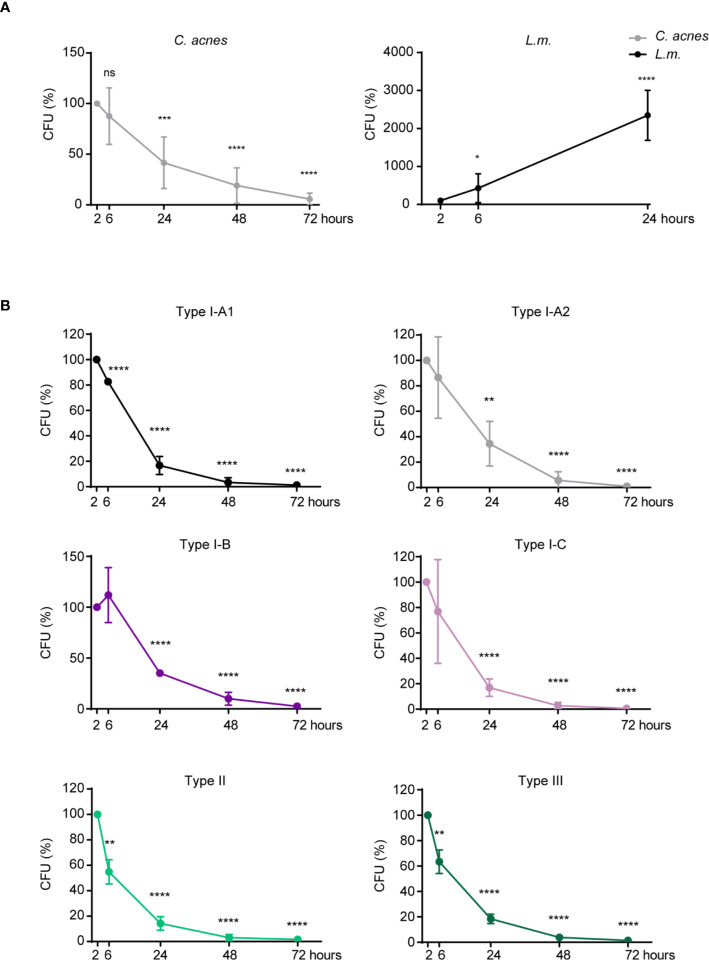
Intracellular degradation of *C. acnes* by human macrophages. A CFU assay was performed using PMA-differentiated THP-1 cells. **(A)** THP-1 cells were either infected with *C. acnes* strain NCTC737 or *L.m.* for indicated timepoints. Graphs show the standard deviation and mean of five independent experiments. **(B)** Differentiated THP-1 cells were infected with one strain of each *C. acnes* phylotype: NCTC737 (phylotype IA_1_), P. acn31 (phylotype IA_2_), KPA171202 (phylotype IB), PV66 (phylotype IC), ATCC11828 (phylotype II), and Asn12 (phylotype III) for indicated timepoints. Graphs show the standard deviation and mean of three independent experiments. **(A, B)** Unpaired t-test of log transformed values compared to the 2-h timepoint was calculated for each timepoint. P-values (*P ≤ 0.05; **P ≤ 0.01; ***P ≤ 0.001; ****P ≤ 0.0001; ns, not significant).

In order to analyze the ability of *C. acnes* to survive and/or replicate inside of its host cell, we performed a CFU assay with *C. acnes*, as well as with the Gram-positive bacteria *L.m.* whose intracellular fate is well studied (32–34). *L.m.* is an invasive bacterium that escapes the host phagosome using the pore-forming toxin listeriolysin O (LLO). Recent literature suggests that *C. acnes* may have developed strategies to neutralize phagosomes in order to survive or even leave the phagosome without being targeted for degradation by bactericidal effector mechanisms (35). For direct comparison, we included a mutant strain of *L.m.* (Δ*hly*, lacking LLO) that is rapidly degraded by the host cell. Indeed, while the load of wildtype *L.m.* increased immensely over a period of 24 h (**Figure 1A**, **Supplementary Figure 1A**), survival and/or propagation of the Δ*hly* strain was prevented by both THP-1 and U-937 cells in a similar time period (**Supplementary Figure 1B**). *C. acnes* reference strain NCTC737 (phylotype IA_1_) on the other hand showed results comparable to the LLO mutant strain suggesting that THP-1 and U-937 cells are indeed able to control *C. acnes* infections (**Figure 1A**, **Supplementary Figure 1A**). A similar pattern could be observed after infection with strains representing each *C. acnes* phylotype, suggesting a strain-independent host mechanism for *C. acnes* degradation in THP-1 cells (**Figure 1B**).

### *C. acnes* Stimulates an IFN-I Response in Human Macrophages

**Figure 2:**
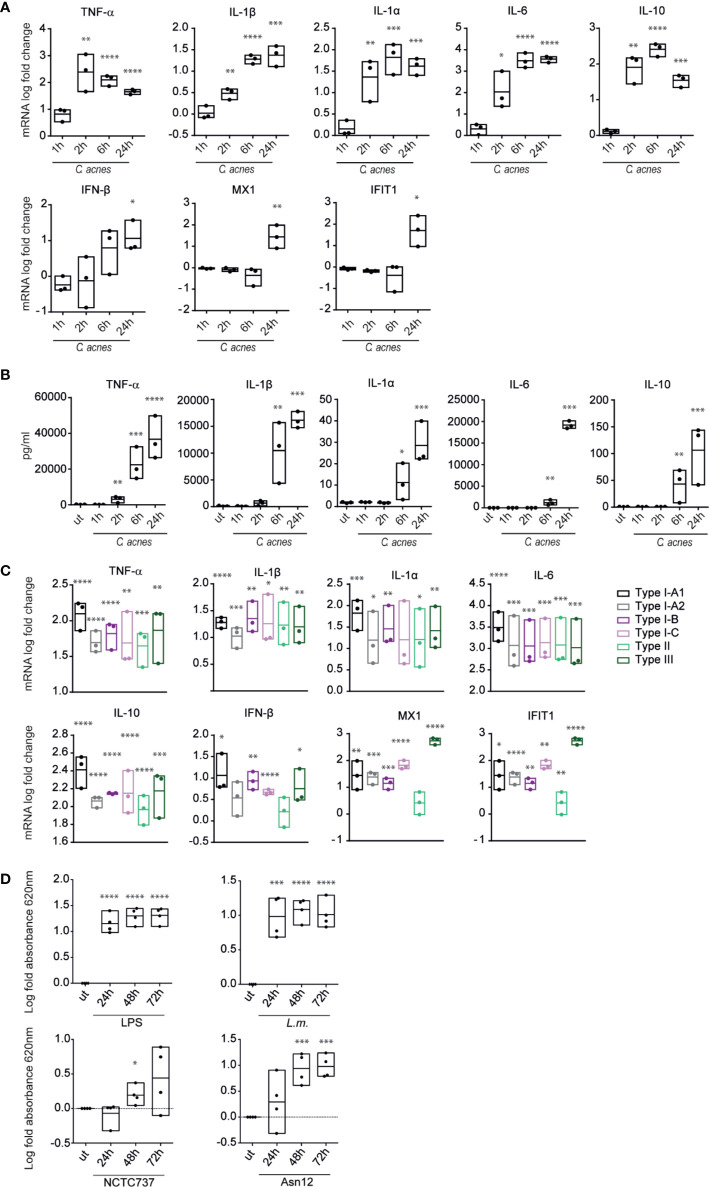
Cytokine expression and secretion in response to *C. acnes*. **(A)** Differentiated THP-1 cells were infected with *C. acnes* strain NCTC737 for indicated timepoints. HPRT-normalized gene expression was measured using RT-qPCR and shown as log transformed fold-change to the uninfected sample. **(B)** Cytokine levels in the supernatant of **(A)** were measured using the Luminex assay. **(C)** Differentiated THP-1 cells were infected with one strain of each *C. acnes* phylotype: NCTC737 (phylotype IA_1_), P. acn31 (phylotype IA_2_), KPA171202 (phylotype IB), PV66 (phylotype IC), ATCC11828 (phylotype II) and Asn12 (phylotype III). HPRT-normalized gene expression was measured using RT-qPCR and shown as log transformed fold change to the uninfected sample. TNF-α, IL-1β, IL-1α, IL6, and IL-10 mRNA expression was measured after 6 h post infection (pi). IFN-β, MX1, and IFIT1 mRNA expression was measured 24 h pi. **(D)** THP1-BlueTM ISG cells (see text) were infected with *L.m., C. acnes* strain NCTC737 and Asn12 or stimulated with 0.4 µg/ml LPS. After 24, 48, and 72 h, supernatant was analyzed by measuring the absorbance at 620 nm with QUANTI-Blue™ solution. **(A–D)** Data represent the mean values of three independent experiments. P. values were calculated using the unpaired t-test of log transformed values compared to the uninfected sample (*P ≤ 0.05; **P ≤ 0.01; ***P ≤ 0.001; ****P ≤ 0.0001).

We characterized the innate response to *C. acnes* infection by determining the production of typical innate cytokines by PMA-differentiated human THP-1 macrophages (**Figure 2**). *C. acnes* strain NCTC737 (phylotype IA_1_) induced the expression of pro-inflammatory (TNF-α, IL-1β, IL-1α and IL-6) and anti-inflammatory (IL-10) cytokines (**Figure 2A**). Furthermore, *C. acnes*-infected THP-1 cells showed an increased secretion of IL-1β protein, confirming *C. acnes*-induced inflammasome activation as published by Qin et al. (**Figure 2B**) (28). The induction of inflammatory cytokines at the mRNA level correlated with the secreted protein level in the supernatant of infected cells. Interestingly, alongside a strong pro-inflammatory cytokine response, we observed an elevated expression of IFN-β as well as type I IFN-stimulated genes (ISGs), such as Interferon-induced GTP-binding protein Mx1 (MX1) and Interferon-induced protein with tetratricopeptide repeats 1 (IFIT1) in *C. acnes* infected THP-1 cells in the delayed phase of infection. This indicates that *C. acnes* not only activates an inflammatory response in human macrophages, but in addition triggers a type I IFN signature.

Comparing the cytokine response caused by infection with different strains of *C. acnes* (one strain of each phylotype) we did not observe any major differences in the amounts of inflammatory cytokine mRNAs. In contrast, the IFN-I-induced genes *MX1* and *IFIT1* differed vastly between strains (**Figure 2C**). Especially *C. acnes* strain Asn12 (phylotype III), which has been recently associated with the skin disease macular hypomelanosis (36), was remarkably potent in inducing ISG transcription. Thus, although *C. acnes* induced inflammatory cytokine response seems to be strain independent, the induction of ISGs differs when comparing various phylotypes.

Among the induced cytokines of **Figure 2B**, IL-6 is controlled by both NFkB and IFN-I pathways (37), suggesting a potential contribution of IFN-I to cytokine secretion during *C. acnes* infection. To determine whether IFN-I activity alone suffices for the detection of secreted gene products we used THP1-Blue™ ISG cells that monitor the interferon signaling pathway and its master transcription factor, the ISGF3 complex, through the stable integration of a secreted embryonic alkaline phosphatase (SEAP) reporter. Specifically, the reporter gene is under an ISG54 minimal promoter with five IFN-stimulated response elements (ISREs). Secreted SEAP can be detected by QUANTI-Blue™ colorimetric enzyme assay. While stimulation with LPS and infection with *L.m.* showed high IFN-induced SEAP levels in the supernatant of cells infected for both 24–72 h, absorbance increased over time with *C. acnes* strain Asn12 (**Figure 2D**). In line with the mRNA expression of ISGs and IFN-β, cells infected wth *C. acnes* strain NCTC737 released significantly smaller quantities of SEAP.

**Figure 3:**
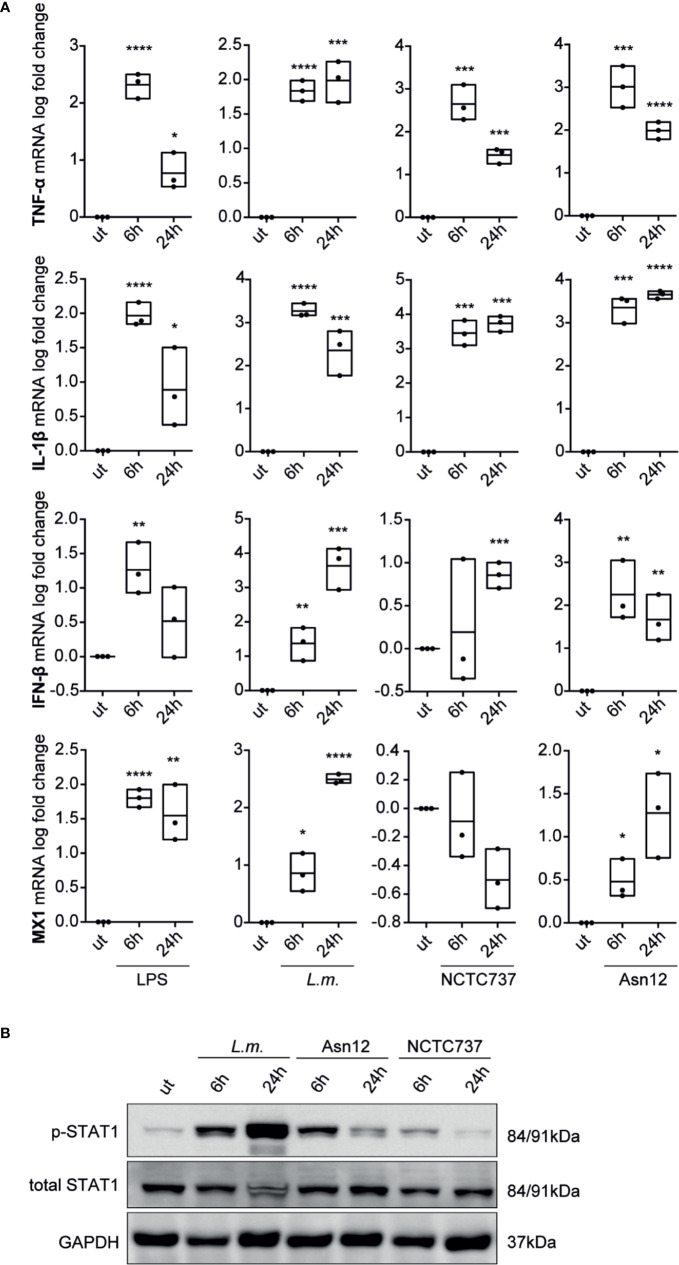
Cytokine expression and type-I IFN signaling in primary monocyte-derived macrophages. Monocyte-derived macrophages were infected with either *C. acnes* strain NCTC737 and Asn12, *L.m.* or stimulated with LPS for either 6 or 24 h. **(A)** HPRT-normalized gene expression of TNF-α, IL-1β, MX1, and IFN-β was measured using RT-qPCR and shown as log transformed fold change to the uninfected sample. Data represent the mean values of three independent experiments. P. values were calculated using the unpaired t-test of log transformed values (*P ≤ 0.05; **P ≤ 0.01; ***P ≤ 0.001; ****P ≤ 0.0001). **(B)** Protein expression of total STAT1, Phospho-Y701 STAT1 and GAPDH (housekeeping gene) was measured by Western blot.

To corroborate our findings with data from primary cells, we analyzed the expression of pro-inflammatory cytokines and type-I IFN signaling in primary monocytes from three different donors. While mRNA expression of the pro-inflammatory cytokine TNF-α and IL-1β were greatly induced upon infection with either *L.m.* or *C. acnes* strains NCTC737 or Asn12 as well as upon stimulation with LPS, the induction of a type-I IFN response was only upregulated upon LPS treatment which could neither be seen with *L.m.* nor with any of the two *C. acnes* strains used (**Supplementary Figure S2A**). On the other hand, M-CSF-differentiated monocyte-derived macrophages (MDMs) from the same donors strongly induced the expression of IFN-β as well as MX1 in addition to a robust inflammatory cytokine response shown by elevated mRNA expression of TNF-α and IL-1β (**Figure 3A**). In addition, infection of primary MDMs with *L.m.* induced the phosphorylation of STAT1 on Tyr701at 6 and 24 h post infection (**Figure 3B**). In line with the mRNA expression of IFN-β as well as MX1, we observed a strong phosphorylation of STAT1 at Tyr701 with *C. acnes* strain Asn12 and a much weaker signal with NCTC737.

As *C. acnes* is a commensal of the human skin, we were interested in the inflammatory cytokine response as well as the IFN-I signature in human keratinocytes. Thus, we infected the human keratinocyte cell line HaCaT with either *L.m.*, *C. acnes* strain Asn12 or stimulated them with LPS for 48 h. Compared to macrophages, HaCaT cells showed only a minor induction of cytokine expression upon infection, even with our positive controls *L.m.* and LPS (**Supplementary Figure S2B**). Although *L.m.* as well as Asn12 induced mRNA expression of TNF-α, IL-6, MX1 and IFIT1, the levels were only slightly above the basal state. Taken together, differentiated THP-1 cells as well as primary human macrophages induce an IFN-I signature as well as a strong inflammatory response upon infection with *C. acnes*.

### Multiple Intracellular Pathways Contribute to Cytokine Production by *C. acnes*-Infected Cells

**Figure 4:**
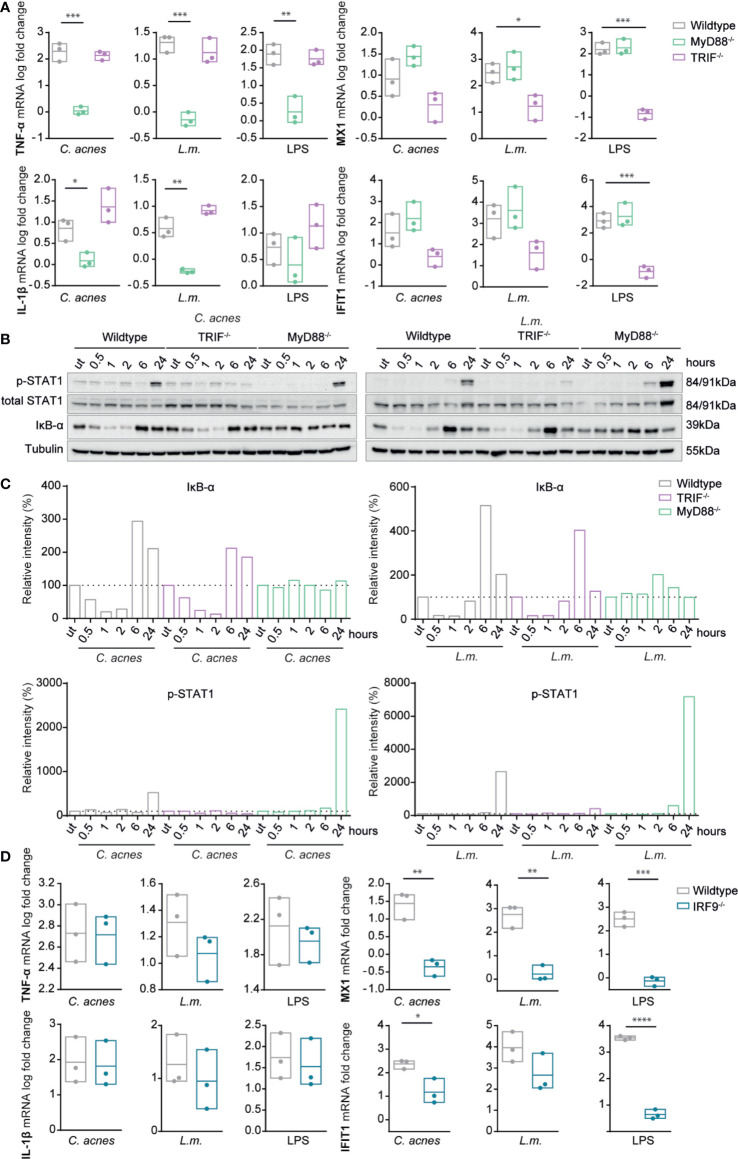
Induction of innate immune signaling pathways in response to *C. acnes* infection in TRIF, MyD88 and IRF9 deficient human macrophages. **(A)** Differentiated wildtype, MYD88-deficient and TRIF-deficient THP-1 cells were infected with either *C. acnes* strain NCTC737, *L.m.* or stimulated with lipopolysaccharide (LPS) for either 6 (TNF-α, IL-1β) or 24 h (MX1, IFIT1). HPRT-normalized gene expression was measured using RT-qPCR and shown as log transformed fold change to the uninfected sample. **(B)** Differentiated wildtype, MYD88-deficient and TRIF THP-1 cells were infected with either *C. acnes* strain NCTC737 or *L.m.* for indicated timepoints. Protein expression of IκB-α, total STAT1, Phospho-Y701 STAT1 and tubulin (housekeeping gene) was measured by Western blot. **(C)** The representative blots in **(B)** were quantified using Image Lab. Relative intensities of the bands were normalized to their corresponding tubulin levels. Data represent relative intensities in percent to the corresponding uninfected control (equals 100%). **(D)** Differentiated wildtype and *IRF9*^-/-^ THP-1 cells were infected with either *C. acnes* strain NCTC737, *L.m.* or stimulated with lipopolysaccharide (LPS) for either 6 (TNF-α, IL-1β) or 24 h (MX1, IFIT1). HPRT-normalized gene expression was measured using RT-qPCR and shown as log transformed fold change to the uninfected sample. **(A, D)** Data represent the mean values of three independent experiments. P. values were calculated using the unpaired t-test of log transformed values (*P ≤ 0.05; **P ≤ 0.01; ***P ≤ 0.001; **** P ≤ 0.0001).

Cytokine expression in infected cells is initiated by signals from cell surface or endosomal TLRs as well as cytoplasmic nucleic acid receptors and inflammasomes (21). We sought to determine the signals targeting inflammatory cytokines and IFN-I in *C. acnes*-infected cells by studying cells deficient for either myeloid differentiation primary response 88 (MyD88) or TRIF (**Figures 4A–C**, **Supplementary Figure S3A**), the eponymous adapters for the major pathways downstream of TLR. Recent experimental evidence shows that *C. acnes* is recognized by a TLR2/6 heterodimer in human keratinocytes (29), which signals *via* the adapter molecule MyD88 to stimulate the expression of inflammatory cytokines. Consistent with this, a knockout of MyD88 in THP-1 cells completely abolished the expression of the inflammatory cytokines TNF-α and IL-1β upon infection with *C. acnes*, *L.m.*, or LPS (**Figure 4A**). In line with the mRNA expression of inflammatory cytokines, cells lacking MyD88 were not able to degrade IκB-α, the inhibitor of the transcription factor nuclear factor ‘kappa-light-chain-enhancer’ of activated B-cells (NF-κB), upon infection with either *C. acnes* or *L.m.* (**Figures 4B, C**). Deletion of TRIF slightly reduced TNF-α mRNA synthesis, but strongly increased that of IL-1β. This suggests a negative feed-back of the TRIF pathway on IL-1β mRNA production through the MyD88 pathway.

Unlike the inflammatory cytokines, IFN-I-dependent STAT1 phosphorylation and ISG induction by either *C. acnes* or *L.m.* remained largely unaffected by the absence of MyD88. In stark contrast, the knockout of adapter molecule TRIF strongly reduced ISG induction and STAT1 Tyr701 phosphorylation upon infection with *C. acnes* or *L.m.* (**Figures 4A–C**). This finding was surprising given the complete independence of *L.m.*-induced IFN-I production in mouse bone marrow-derived macrophages (38). To examine whether the involvement of TRIF reflected signaling through a non-canonical, endosomal TLR2-TRIF pathway as recently observed in mouse peritoneal macrophages infected with *L.m.* (22), we infected THP-1 cells with the escape deficient *L.m.* strain Δ*hly*. LLO-deficient *L.m.* were still capable of inducing the pro-inflammatory response *via* the TLR2 axis (TNF-α and IL-1β mRNA), however, they were unable to stimulate IFN-I signaling as shown by the mRNA expression of MX1 and IFIT1 (**Figure S3B**). This result suggests that *L.m.*-induced ISG expression in THP-1 cells is independent of TLR-TRIF signaling, however, dependent on the adapter TRIF in a different signaling context.

IFN-I exert gene control *via* canonical and noncanonical mechanisms (39). The canonical mode requires formation of the interferon-stimulated gene factor 3 (ISGF3) complex, a trimeric protein complex consisting of STAT1, STAT2 and IRF9. We used IRF9 knockout THP-1 cells to examine whether the expression of MX1 and IFIT1 was induced by the canonical IFN-I signaling pathway in *C. acnes-*infected cells. Indeed, a knockout of IRF9 completely abolished the expression of ISGs upon infection with *C. acnes* whereas it did not affect the proinflammatory cytokine response (**Figure 4D**, **Supplementary Figure S3C**). Thus, *C. acnes* induced IFN-I signaling occurs *via* the canonical type I IFN pathway.

### *C. acnes*-Induced Type I IFN Signaling Requires a cGAS-STING Pathway

To further interrogate the role of TRIF in IFN-I production, we investigated the role of STING and its upstream regulator cGAS. These studies were in accordance with a recent report demonstrating a TRIF requirement for STING signaling in cells infected with herpes simplex virus (40).

**Figure 5:**
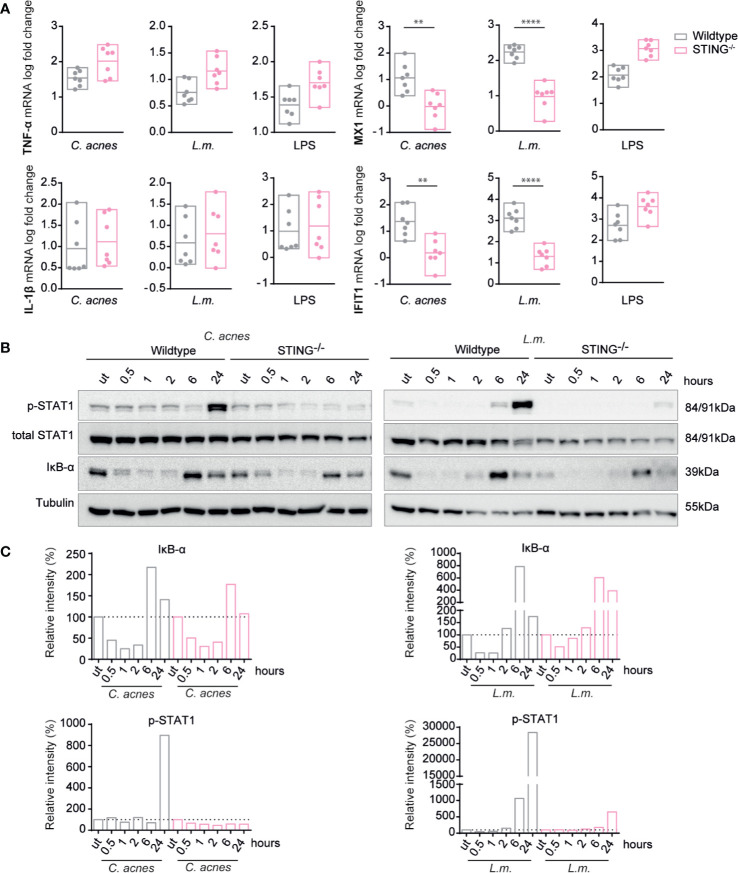
Induction of innate immune signaling pathways in response to *C. acnes* infection in STING-deficient human macrophages. **(A)** Differentiated wildtype and STING-deficient THP-1 cells were infected with either *C. acnes* strain NCTC737, *L.m.* or stimulated with lipopolysaccharide (LPS; 0.4µg/ml) for either 6 (TNF-α, IL-1β) or 24 h (MX1, IFIT1). HPRT-normalized gene expression was measured using RT-qPCR and shown as log transformed fold change to the uninfected sample. Data represent the mean values of seven independent experiments. P values were calculated using the unpaired t-test of log transformed values (**P ≤ 0.01; ****P ≤ 0.0001). **(B)** Differentiated wildtype and STING-deficient THP-1 cells were infected with either *C. acnes* strain NCTC737 or *L.m.* for indicated timepoints. Protein expression of IκB-α, total STAT1, Phospho-Y701 STAT1, and tubulin (housekeeping gene) was measured by western blot. **(C)** The representative blots in **(B)** were quantified using Image Lab. Relative intensities of the bands were normalized to their corresponding tubulin levels. Data represent relative intensities in percent to the corresponding uninfected control (equals 100%).

Unlike wildtype cells, ISG induction in response to infection with either *C. acnes* or *L.m.* was not detected in THP-1 cells lacking STING (**Supplementary Figure S4A**, **Figure 5A**). The complete lack of a response to IFN-I confirms the absence of TLR2-TRIF signaling, which is independent of STING. As expected, IFN signaling in LPS-stimulated cells was independent of STING signaling as we observed a slight increase in ISG expression. These results were corroborated by showing that the phosphorylation of STAT1 on tyrosine 701 is strongly reduced in both *C. acnes* and *L.m.*-infected cells lacking the adapter STING (**Figures 5B, C**). In contrast, the degradation of the NF-κB inhibitor IκB-α in wildtype and knockout cells was similar. These results demonstrate that *C. acnes* induced type I IFN signaling is dependent on the adapter STING, which is positively regulated by the adapter TRIF.

**Figure 6:**
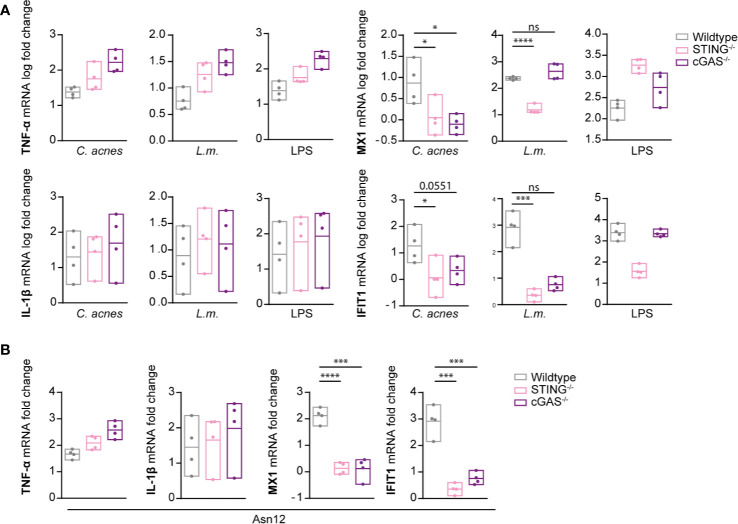
Induction of innate immune signaling pathways in response to *C. acnes* infection in cGAS-deficient human macrophages. **(A)** Differentiated wildtype, STING-deficient and cGAS-deficient THP-1 cells were infected with either *C. acnes* strain NCTC737, *L.m.* or stimulated with lipopolysaccharide (LPS; 0.4 µg/ml) for either 6 (TNF-α, IL-1β) or 24 h (MX1, IFIT1). **(B)** Differentiated wildtype, STING-deficient and cGAS-deficient THP-1 cells were infected with either *C. acnes* strain Asn12, *L.m.* or stimulated with lipopolysaccharide (LPS; 0.4 µg/ml) for either 6 (TNF-α, IL-1β) or 24 h (MX1, IFIT1). **(A, B)** HPRT-normalized gene expression was measured using RT-qPCR and shown as log transformed fold change to the uninfected sample. Data represent the mean values of four independent experiments. P values were calculated using the unpaired t-test of log transformed values (*P ≤ 0.05; ***P ≤ 0.001; ****P ≤ 0.0001).

When infected with *C. acnes*, cGAS-deficient THP-1 cells revealed an essential function of the DNA sensor for the induction of ISG expression, very similar to the STING knockouts (**Figure 6A**). *L.m.* on the other hand was capable of ISG induction in absence of cGAS. This is in accordance with published data showing that *L.m.* is able to stimulate STING in a cGAS-independent manner (26, 27). Interestingly, cGAS independence was much less pronounced when IFN-b synthesis was measured at earlier time points (23). This raises the possibility that cyclic di-nucleotides produced by *L.m.* accumulate to generate cGAS-independence of STING activation at delayed stages of infection.

**Figure 7:**
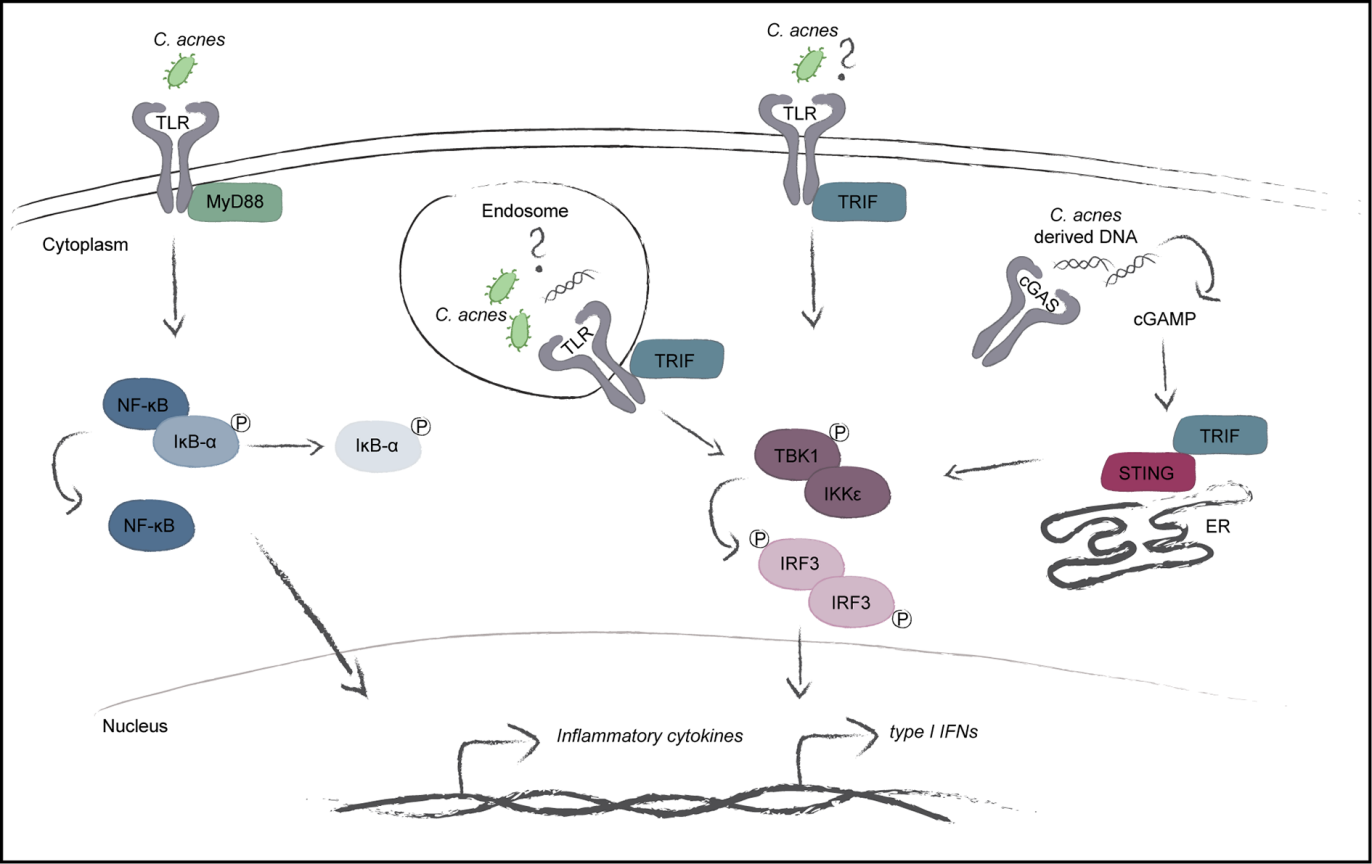
Model of the *C. acnes*-induced intracellular signaling pathways. *C. acnes* recognition in human macrophages is carried out by membrane bound toll-like receptors with its adapter MyD88. This further leads to the activation of the NF-κB pathway and the subsequent expression of inflammatory cytokines. The TLR adapter TRIF shows importance during *C. acnes*-induced IFN-signaling. TRIF can be activated by specific TLR activation on the cell membrane or in the endosome and has also been shown to positively regulate the cGAS/STING pathway. Whether *C. acnes* is recognized by membrane-bound or endosomal TLR-TRIF or simply boosts the cGAS/STING pathway in a TRIF-dependent manner is not known.

In order to determine whether IFN signaling *via* the cGAS/STING pathway is generally employed by *C. acnes* to stimulate an IFN-I response independently of the phylotype, we performed infection experiments with *C. acnes* strain Asn12, the most potent IFN-I inducer (**Figure 2C**), in cells lacking either cGAS or STING. As depicted in **Figure 6B**, *C. acnes* strain Asn12 strongly induced ISG expression in wildtype cells. However, ISG expression was lost in cells lacking either cGAS or STING. Taken together our results suggest TRIF-dependent cGAS-STING signals in a default pathway of IFN synthesis in *C. acnes*-infected human macrophages (**Figure 7**).

### *C. acnes* Intracellular Degradation Is Independent of Inflammatory Cytokine and Type I IFN Response

**Figure 8:**
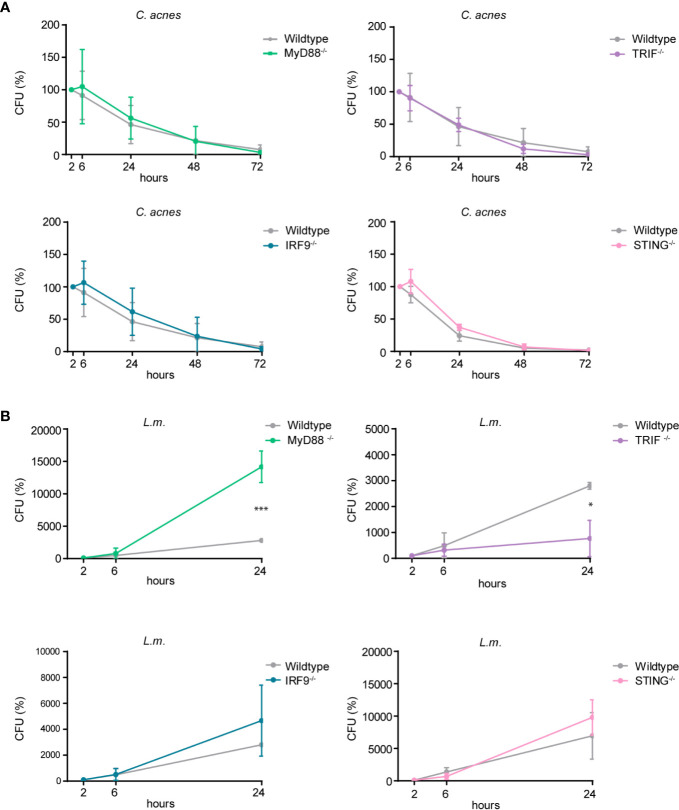
Intracellular survival of *C. acnes* and *L.m.* in human macrophages deficient for either MyD88, TRIF, IRF9, or STING. Colony-forming-unit (CFU) assay was performed using PMA-differentiated THP-1 cells deficient for either MyD88, TRIF, IRF9, or STING. THP-1 cells were either infected with *C. acnes* strain NCTC737 **(A)** or *L.m.* **(B)** for indicated timepoints. Graphs show the standard deviation and mean of three independent experiments. Unpaired t-test of log transformed values was calculated. P-values (*P ≤ 0.05).

Cytokines shape the local inflammatory environment of a cell, but they can also influence the establishment of cell-autonomous immunity. To determine whether pro-inflammatory cytokine and/or interferon responses impact on effector mechanisms that influence bacterial growth we performed CFU assays with wildtype cells or cells lacking either MyD88, TRIF, STING or the ISGF3 subunit IRF9. As depicted in **Figure 8A**, *C. acnes* recovery from infected THP-1 cells did not significantly differ when comparing wildtype versus any of the knockout cells. Contrary to *C. acnes*, the intracellular growth of *L.m.* was curtailed by the MyD88-dependent proinflammatory response (**Figure 8B**). This differs from results obtained in mouse bone marrow-derived macrophages (41). Surprisingly, the knockout of TRIF reduced the growth of *L.m.* whereas such an effect was not observed when IFN-I synthesis or response was abrogated by the knockouts of STING or IRF9. This suggests that an IFN-independent activity of TRIF blunts the ability of THP-1 cells to restrict *L.m.* growth. Taken together, our data show that reduced proinflammatory cytokine or IFN-I synthesis fails to make macrophages permissive for intracellular growth of *C. acnes*, and that the TLR adaptors MyD88 and TRIF impinge on *L.m.* replication in opposing manners.

## Discussion

Infection with *C. acnes* is associated with a variety of diseases ranging from inflammatory acne lesions of the skin to prostate disease, soft-tissue infections and sarcoidosis. The latter disease, characterized by bouts of inflammation in a variety of different organs, has been genetically correlated with single-nucleotide polymorphism (SNPs) in the nucleotide binding domains of the genes encoding the intracellular pattern recognition receptor NOD1 (42). The deposition and survival of *C. acnes* in internal organs was proposed to result from its ability to survive in circulating monocytes and epithelial cells [reviewed in (43)]. The pathogenesis of the human skin disorder acne vulgaris is believed to occur from altered keratinization (44), as well as elevated sebum production (45), leading to proliferation of *C. acnes* in the human hair follicle (15, 46). While the association of *C. acnes* with acne (3) and post-surgical implant infection (6–10) is well-described, its intracellular fate and means of inducing innate immune signaling pathways is still not fully understood.

It has previously been shown that *C. acnes* induces a strong inflammatory response *in vitro* from THP-1, as well as U937 monocytes (12), which is consistent with data showing that inflammatory acne lesions have an elevated expression of cytokines such as IL-1β, TNF-α and IL-10 compared to uninvolved adjacent skin (47). We now extend these studies to primary human monocytes as well as monocyte-derived macrophages. The data clearly show that the ability to induce IFN-I synthesis is a property of differentiated macrophages much more than of their monocyte progenitors. As *C. acnes* is a Gram-positive bacterium, several studies have focused on the recognition of the *C. acnes*’ peptidoglycan (PG) layer by intracellular NOD receptors (42) and membrane-bound Toll-like receptors, particularly TLR2, 1 and 6 (29, 48, 49). While there is a clear understanding that *C. acnes* triggers inflammatory cytokine expression *via* heterodimers of TLR2 with TLR1 or 6, followed by the activation of the NF-κB as well as mitogen-activated protein kinase (MAPK) signaling cascade (50, 51), it has not been known if, and indeed, how *C. acnes* may induce the interferon signaling pathway. In our studies, *C. acnes* infection of THP-1 cells lacking the TLR adapter MyD88 clearly showed that production of inflammatory cytokines, but not that of IFN-I, was driven by a MyD88 pathway. This is perfectly in line with current literature suggesting a TLR2, 1 and 6 dependency.

To our surprise, we observed that the interferon response in a *C. acnes* infection strongly relied on the adapter TRIF. This contradicts the notion that TRIF pathways are downstream of TLRs 3 and 4, but not those recognizing ligands of Gram-positive bacteria (52). Infection of differentiated THP-1 cells with the Gram-positive bacterium *L.m.* showed similar dependency on TRIF for IFN-I synthesis. The recognition of *L.m.* seems to strongly depend on the macrophage population, as the IFN-I production in mouse bone marrow-derived macrophages shows complete independence of TRIF (38) while macrophages residing the mouse peritoneum show IFN-I synthesis *via* a TLR2-TRIF pathway upon *L.m.* infection (22). Therefore, one possible interpretation of our results would be the wiring of a TLR2-TRIF pathway in human macrophages. As for *L.m.* infections in THP-1 cells, a TLR2-TRIF pathway for IFN-I synthesis can be ruled out due to the lack of ISG expression upon the infection with the escape-deficient *hly* mutant strain which has been shown in this study. However, owing to our finding of a drastic reduction of the response to IFN-I in either TRIF, cGAS and STING knockout cells, we posit an association between the cGAS-STING pathway and TRIF as proposed by Wang et al. (40). These authors provide data showing that TRIF is needed for STING dimerization and is, therefore, required for STING-mediated transcriptional activation of IRF3 homodimers. Thus, our data would be the first to extend this signaling mechanism to a bacterial pathogen. Whereas direct interaction between TRIF and STING was demonstrated, an involvement of TLR in this interaction was not formally ruled out.

We noted an enhanced expression of pro-inflammatory cytokine mRNAs, especially those for TNF-α, in knockouts of either STING or cGAS. This increase was not observed in cells deficient for the ISGF3 subunit IRF9, suggesting it is not result of a transcription-based cooperativity between IRF and STAT transcription factor families. A possible explanation is provided by a report from Clark et al. (53) showing crosstalk between the canonical IκB kinase (IKK) complex (IKKα-IKKβ) and the TANK-binding kinase 1 (TBK1)-IKKϵ kinases which negatively regulates the canonical IKKs. Consequently, by blocking the TBK1-IKKϵ axis downstream of STING activation one would lose the negative regulation of the canonical IKKs and would, therefore, enhance the inflammatory response.

Our data showed that different *C. acnes* phylotypes differ significantly in their ability to produce a response to IFN-I, however they show similar patterns in expression of pro-inflammatory cytokines. This can be explained by the variations in morphological characteristics such as cell wall composition, production of different virulence factors and other immunologically relevant properties of the different *C. acnes* phylotypes (2). This raises the possibility that inflammatory diseases associated with these phylotypes are more prone to the proinflammatory effects, such as the synthesis of chemokines or the enhanced synthesis of some inflammatory mediators such as nitric oxide (NO) (18).

We did not find any evidence of prolonged *C. acnes* survival in infected THP-1 or U937 cells (**Figure 1** and **Supplementary Figure S1**). In fact, the loss of viable bacteria corresponded well to that of the escape-deficient *hly* mutant of *L.m.* This suggests that unlike wildtype *L.m*, *C. acnes* is not endowed with a widely applicable mechanism for intracellular survival. Our data do not rule out, however, that some primary cell types might present niches for intracellular survival and organismic spread. In contrast to our data, Fischer *et al*. showed a persistence of *C. acnes* in THP-1 cells over a period of 3 days (35). They suggested that *C. acnes* can either neutralize the phagosomal acidic pH or escape the phagosome in order to prevent its degradation, the latter being in line with our data showing *C. acnes*-induced cGAS-STING signaling. However, while using the same host cell, Fischer *et al*. used a very low infection rate, different bacterial strains, as well as different antibiotic treatments to kill extracellular bacteria, which could explain the contrasting results. On the other hand, Nakamura *et al*. discovered that *C. acnes* can be taken up by autophagic vacuoles, especially when using high infection rates (54). While *C. acnes* could persist in those vacuoles in HeLa cells after 3 days of infection, the loss of bacteria over time in either the murine macrophage cell line RAW264.7 or mouse embryonic fibroblasts (MEFs) suggests a cell type specificity for *C. acnes* intracellular degradation. Interestingly, type-I IFN signaling does not restrict *C. acnes* intracellular growth and/or host-induced bacterial killing as seen in **Figure 1B**. Not even the most potent strain in inducing an IFN-I axis, Asn12, showed differences in bacterial numbers counted by CFU.

Our data provide convincing evidence that *C. acnes* activates the cGAS-STING pathway and that, unlike *L.m.*, does not secrete cyclic di-nucleotides capable of STING activation. An important open question to be addressed in future research is how *C. acnes* DNA acquires access to the intracellular sensor cGAS. This could occur either through phagosome leakage or through a cytoplasmic escape mechanism that is not necessarily associated with intracellular growth. Another open question concerns the consequences of an IFN response during *C. acnes* infection, both for local and systemic inflammation. Taken together, studying the innate immune signaling cascades activated downstream of *C. acnes* recognition is an important step in order to fully understand pathogenic characteristics. This information can further be used for pathogen-specific novel therapies against diseases where bacterial accumulation plays an essential role.

## Data Availability Statement

All datasets presented in this study are included in the article/**Supplementary Material**.

## Author Contributions

KF performed experiments, evaluated the data and wrote the manuscript. RT and AP designed the study. SC and AM provided genetically engineered cells and bacterial strains. SS isolated primary PMBC. TD designed the study and wrote the manuscript. All authors contributed to the article and approved the submitted version.

## Funding

This work was funded by the Austrian Science Fund through grants P 25186-B22 and SFB F6103. KF is an associate member of the SMICH doctoral school funded by the Austrian Science Fund.

## Conflict of Interest

Authors SS and SC were employed, by Vienna and Boehringer Ingelheim, Vienna. Author RT was employed by the company Themis Bioscience, Vienna, Austria.

The remaining authors declare that the research was conducted in the absence of any commercial or financial relationships that could be construed as a potential conflict of interest.

## Supplementary Material

Supplementary Figure 1 **(A)** CFU assay was performed using PMA-differentiated U-937 cells. U-937 cells were infected with either *C. acnes* strain NCTC737 (left; grey) or wildtype *L.m.* (right; black) for indicated timepoints. **(B)** CFU assay was performed using PMA-differentiated THP-1 (black squares) and U-937 (grey triangles) cells. Cells were infected with the *L.m.* mutant strain Δ*hly* for indicated timepoints. **(A–B)** Representative of two independent experiments.

Supplementary Figure 2 **(A)** Primary monocytes were infected with either *C. acnes* strain NCTC737 and Asn12, *L.m.* or stimulated with LPS for either 6 or 24 h. HPRT-normalized gene expression of TNF-α, IL-1β, MX1 and IFIT1 was measured using RT-qPCR and shown as log transformed fold change to the uninfected sample. **(B)** HaCaT cells were infected with either *C. acnes* strain Asn12, *L.m.* or stimulated with LPS for 48 h. HPRT-normalized gene expression of TNF-α, MX1, IFIT1 and IL-6 was measured using RT-qPCR and shown as log transformed fold change to the uninfected sample. **(A–B)** Data represent the mean values of three independent experiments. P. values were calculated using the unpaired t-test of log transformed values (*P ≤ 0.05; **P ≤ 0.01; ***P ≤ 0.001).

Supplementary Figure 3 **(A)** Confirmation of CRISPR/Cas9 generated MyD88 and TRIF knockout THP-1 cells. MyD88, TRIF and tubulin protein levels of wildtype, MyD88 and TRIF knockout THP-1 cells was analyzed by western blot. **(B)** Differentiated THP-1 cells were infected with either *C. acnes* strain NCTC737, wildtype *L.m.* or LLO-deficient *L.m.* strain Δ*hly* for either 6 (TNF-α, IL-1β) or 24 h (MX1, IFIT1). HPRT-normalized gene expression was measured using RT-qPCR and shown as log transformed fold change to the uninfected sample. Data represent the mean values of three independent experiments. P. values were calculated using the unpaired t-test of log transformed values (*P ≤ 0.05; **P ≤ 0.01; P***≤0.001). **(C)** Confirmation of CRISPR/Cas9 generated IRF9 knockout THP-1 cells. IRF9 protein level of wildtype and IRF9 knockout THP-1 cells was analyzed by western blot.

Supplementary Figure 4 **(A)** Confirmation of STING knockout THP-1 cells. STING and tubulin protein levels of wildtype and STING knockout THP-1 cells was analyzed by western blot. **(B)** Confirmation of cGAS knockout THP-1 cells. STING, cGAS and tubulin protein levels of wildtype, cGAS and STING knockout THP-1 cells was analyzed by western blot.

## References

[1] Grice EA Segre JA The skin microbiome. Nat Rev Microbiol (2011) 9:244–53. 10.1038/nrmicro2537 PMC3535073 21407241

[2] McDowell A Nagy I Molecular Medical Microbiology. Part 7 Localized Infect (2015) 1:837–58. 10.1016/b978-0-12-397169-2.00046-9

[3] Taylor M Gonzalez M Porter R Pathways to inflammation: acne pathophysiology. Eur J Dermatol (2011) 21:323–33. 10.1684/ejd.2011.1357 21609898

[4] Davidsson S Mölling P Rider JR Unemo M Karlsson MG Carlsson J Frequency and typing of Propionibacterium acnes in prostate tissue obtained from men with and without prostate cancer. Infect Agents Cancer (2016) 11:26. 10.1186/s13027-016-0074-9 PMC4899914 27284286

[5] Cohen RJ Shannon BA McNeal JE Shannon T Garrett KL Propionibacterium acnes associated with inflammation in radical prostatectomy specimens: A possible link to cancer evlution? J Urol (2005) 173:1969–74. 10.1097/01.ju.0000158161.15277.78 15879794

[6] Kadler BK Mehta SS Funk L Propionibacterium acnes infection after shoulder surgery. Int J Shoulder Surg (2015) 9:139–44. 10.4103/0973-6042.167957 PMC4640005 26622132

[7] Pozo JLD Tran NV Petty PM Johnson CH Walsh MF Bite U Pilot study of association of bacteria on breast implants with capsular contracture. J Clin Microbiol (2009) 47:1333–7. 10.1128/jcm.00096-09 PMC2681843 19261794

[8] Rieger UM Pierer G Lüscher NJ Trampuz A Sonication of Removed Breast Implants for Improved Detection of Subclinical Infection. Aesthet Plast Surg (2009) 33:404–8. 10.1007/s00266-009-9333-0 19322605

[9] Piper KE Jacobson MJ Cofield RH Sperling JW Sanchez-Sotelo J Osmon DR Microbiologic diagnosis of prosthetic shoulder infection by use of implant sonication. J Clin Microbiol (2009) 47:1878–84. 10.1128/jcm.01686-08 PMC2691098 19261785

[10] Portillo ME Corvec S Borens O Trampuz A Propionibacterium acnes: an underestimated pathogen in implant-associated infections. BioMed Res Int (2013) 2013:804391. 10.1155/2013/804391 24308006 PMC3838805

[11] Holland C Mak TN Zimny-Arndt U Schmid M Meyer TF Jungblut PR Proteomic identification of secreted proteins of Propionibacterium acnes. BMC Microbiol (2010) 10:230. 10.1186/1471-2180-10-230 20799957 PMC3224659

[12] Vowels BR Yang S Leyden JJ Induction of proinflammatory cytokines by a soluble factor of Propionibacterium acnes: implications for chronic inflammatory acne. Infect Immun (1995) 63:3158–65. 10.1128/iai.63.8.3158-3165.1995 PMC173431 7542639

[13] Bojar RA Holland KT Acne and propionibacterium acnes. Clin Dermatol (2004) 22:375–9. 10.1016/j.clindermatol.2004.03.005 15556721

[14] Kurokawa I Danby FW Ju Q Wang X Xiang LF Xia L New developments in our understanding of acne pathogenesis and treatment. Exp Dermatol (2009) 18:821–32. 10.1111/j.1600-0625.2009.00890.x 19555434

[15] Leeming JP Holland KT Cuncliffe WJ The microbial colonization of inflamed acne vulgaris lesions. Brit J Dermatol (1988) 118:203–8. 10.1111/j.1365-2133.1988.tb01775.x 2964856

[16] McNab F Mayer-Barber K Sher A Wack A O’Garra A Type I interferons in infectious disease. Nat Rev Immunol (2015) 15:87–103. 10.1038/nri3787 25614319 PMC7162685

[17] Isaacs A Lindenmann J Virus interference. I. The interferon. Proc R Soc Lond Ser B Biol Sci (1957) 147:258–67. 10.1098/rspb.1957.0048 26297790

[18] Rauch I Müller M Decker T The regulation of inflammation by interferons and their STATs. Jak-stat (2013) 2:e23820. 10.4161/jkst.23820 24058799 PMC3670275

[19] Decker T Müller M Stockinger S The Yin and Yang of type I interferon activity in bacterial infection. Nat Rev Immunol (2005) 5:675–87. 10.1038/nri1684 16110316

[20] Abbas AK Lichtman AH Pillai S by I Baker DL Cellular and Molecular Immunology. Sect Introd Immune Syst (2010) 6:19–46. 10.1016/b978-1-4160-3123-9.50007-3

[21] Mogensen TH Pathogen recognition and inflammatory signaling in innate immune defenses. Clin Microbiol Rev (2009) 22:240–73. 10.1128/cmr.00046-08 PMC2668232 19366914

[22] Aubry C Corr SC Wienerroither S Goulard C Jones R Jamieson AM Both TLR2 and TRIF contribute to interferon-β production during Listeria infection. PloS One (2012) 7:e33299. 10.1371/journal.pone.0033299 22432012 PMC3303824

[23] Hansen K Prabakaran T Laustsen A Jørgensen SE Rahbæk SH Jensen SB Listeria monocytogenes induces IFNβ expression through an IFI16-, cGAS- and STING-dependent pathway. EMBO J (2014) 33:1654–66. 10.15252/embj.201488029 PMC4194099 24970844

[24] Ahn J Barber GN STING signaling and host defense against microbial infection. Exp Mol Med (2019) 51:1–10. 10.1038/s12276-019-0333-0 PMC6906460 31827069

[25] Chen Q Sun L Chen ZJ Regulation and function of the cGAS–STING pathway of cytosolic DNA sensing. Nat Immunol (2016) 17:1142–9. 10.1038/ni.3558 27648547

[26] Woodward JJ Iavarone AT Portnoy DA c-di-AMP secreted by intracellular Listeria monocytogenes activates a host type I interferon response. Sci New York N Y (2010) 328:1703–5. 10.1126/science.1189801 PMC3156580 20508090

[27] Burdette DL Monroe KM Sotelo-Troha K Iwig JS Eckert B Hyodo M STING is a direct innate immune sensor of cyclic di-GMP. Nature (2011) 478:515–8. 10.1038/nature10429 PMC3203314 21947006

[28] Qin M Pirouz A Kim M-H Krutzik SR Garbán HJ Kim J Propionibacterium acnes Induces IL-1β secretion via the NLRP3 inflammasome in human monocytes. J Invest Dermatol (2013) 134:381–8. 10.1038/jid.2013.309 PMC4116307 23884315

[29] Su Q Grabowski M Weindl G Recognition of Propionibacterium acnes by human TLR2 heterodimers. Int J Med Microbiol (2017) 307:108–12. 10.1016/j.ijmm.2016.12.002 28024924

[30] Platanitis E Demiroz D Schneller A Fischer K Capelle C Hartl M A molecular switch from STAT2-IRF9 to ISGF3 underlies interferon-induced gene transcription. Nat Commun (2019) 10:2921. 10.1038/s41467-019-10970-y 31266943 PMC6606597

[31] Sanjana NE Shalem O Zhang F Improved vectors and genome-wide libraries for CRISPR screening. Nat Methods (2014) 11:783–4. 10.1038/nmeth.3047 PMC4486245 25075903

[32] Portnoy DA Auerbuch V Glomski IJ The cell biology of Listeria monocytogenes infection. J Cell Biol (2002) 158:409–14. 10.1083/jcb.200205009 PMC2173830 12163465

[33] Portnoy DA Chakraborty T Goebel W Cossart P Molecular determinants of Listeria monocytogenes pathogenesis. Infect Immun (1992) 60:1263–7. 10.1128/iai.60.4.1263-1267.1992 PMC256991 1312514

[34] Schnupf P Portnoy DA Listeriolysin O: a phagosome-specific lysin. Microbes Infect (2007) 9:1176–87. 10.1016/j.micinf.2007.05.005 17720603

[35] Fischer N Mak TN Shinohara DB Sfanos KS Meyer TF Brüggemann H Deciphering the intracellular fate of Propionibacterium acnes in macrophages. BioMed Res Int (2013) 2013:603046. 10.1155/2013/603046 23862148 PMC3687600

[36] McDowell A Nagy I Magyari M Barnard E Patrick S The opportunistic pathogen Propionibacterium acnes: insights into typing, human disease, clonal diversification and CAMP factor evolution. PloS One (2013) 8:e70897. 10.1371/journal.pone.0070897 24058439 PMC3772855

[37] Wienerroither S Shukla P Farlik M Majoros A Stych B Vogl C Cooperative Transcriptional Activation of Antimicrobial Genes by STAT and NF-κB Pathways by Concerted Recruitment of the Mediator Complex. Cell Rep (2015) 12:300–12. 10.1016/j.celrep.2015.06.021 PMC4521078 26146080

[38] Stockinger S Reutterer B Schaljo B Schellack C Brunner S Materna T IFN Regulatory Factor 3-Dependent Induction of Type I IFNs by Intracellular Bacteria Is Mediated by a TLR- and Nod2-Independent Mechanism. J Immunol (2004) 173:7416–25. 10.4049/jimmunol.173.12.7416 15585867

[39] Majoros A Platanitis E Kernbauer-Hölzl E Rosebrock F Müller M Decker T Canonical and Non-Canonical Aspects of JAK-STAT Signaling: Lessons from Interferons for Cytokine Responses. Front Immunol (2017) 8:29. 10.3389/fimmu.2017.00029 28184222 PMC5266721

[40] Wang X Majumdar T Kessler P Ozhegov E Zhang Y Chattopadhyay S STING Requires the Adaptor TRIF to Trigger Innate Immune Responses to Microbial Infection. Cell Host Microbe (2016) 20:329–41. 10.1016/j.chom.2016.08.002 PMC5026396 27631700

[41] Edelson BT Unanue ER MyD88-Dependent but Toll-Like Receptor 2-Independent Innate Immunity to Listeria : No Role for Either in Macrophage Listericidal Activity. J Immunol (2002) 169:3869–75. 10.4049/jimmunol.169.7.3869 12244184

[42] Tanabe T Ishige I Suzuki Y Aita Y Furukawa A Ishige Y Sarcoidosis and NOD1 variation with impaired recognition of intracellular Propionibacterium acnes. Biochim Et Biophys Acta Bba Mol Basis Dis (2006) 1762:794–801. 10.1016/j.bbadis.2006.07.006 16935475

[43] Leheste JR Ruvolo KE Chrostowski JE Rivera K Husko C Miceli A acnes-Driven Disease Pathology: Current Knowledge and Future Directions. Front Cell Infect Mi (2017) 7:81:81. 10.3389/fcimb.2017.00081 PMC5348501 28352613

[44] Scott EJV MacCardle RC Keratinization of the Duct of the Sebaceous Gland and Growth Cycle of the Hair Follicle in the Histogenesis of Acne in Human Skin 1. J Invest Dermatol (1956) 27:405–29. 10.1038/jid.1956.115 13406288

[45] Grange PA Weill B Dupin N Batteux F Does inflammatory acne result from imbalance in the keratinocyte innate immune response? Microbes Infect Institut Pasteur (2010) 12:1085–90. 10.1016/j.micinf.2010.07.015 20691803

[46] Burkhart CG Burkhart CN Lehmann PF Classic diseases revisited: Acne: a review of immunologic and microbiologic factors. Postgrad Med J (1999) 75:328–31. 10.1136/pgmj.75.884.328 PMC1741272 10435165

[47] Kang S Cho S Chung JH Hammerberg C Fisher GJ Voorhees JJ Inflammation and Extracellular Matrix Degradation Mediated by Activated Transcription Factors Nuclear Factor-κB and Activator Protein-1 in Inflammatory Acne Lesions in Vivo. Am J Pathol (2005) 166:1691–9. 10.1016/s0002-9440(10)62479-0 PMC1602424 15920154

[48] Kim J Ochoa M-T Krutzik SR Takeuchi O Uematsu S Legaspi AJ Activation of Toll-Like Receptor 2 in Acne Triggers Inflammatory Cytokine Responses. J Immunol (2002) 169:1535–41. 10.4049/jimmunol.169.3.1535 PMC4636337 12133981

[49] Zhang B Choi YM Lee J An IS Li L He C Toll-like receptor 2 plays a critical role in pathogenesis of acne vulgaris. BioMed Dermatol (2019) 3:4. 10.1186/s41702-019-0042-2

[50] Chen Q Koga T Uchi H Hara H Terao H Moroi Y Propionibacterium acnes-induced IL-8 production may be mediated by NF-κB activation in human monocytes. J Dermatol Sci (2002) 29:97–103. 10.1016/s0923-1811(02)00013-0 12088610

[51] Tsai H-H Lee W-R Wang P-H Cheng K-T Chen Y-C Shen S-C Propionibacterium acnes-induced iNOS and COX-2 protein expression via ROS-dependent NF-κB and AP-1 activation in macrophages. J Dermatol Sci (2013) 69:122–31. 10.1016/j.jdermsci.2012.10.009 23178030

[52] Kawasaki T Kawai T Toll-like receptor signaling pathways. Front Immunol (2014) 5:461. 10.3389/fimmu.2014.00461 25309543 PMC4174766

[53] Clark K Takeuchi O Akira S Cohen P The TRAF-associated protein TANK facilitates cross-talk within the IkappaB kinase family during Toll-like receptor signaling. P Natl Acad Sci USA (2011) 108:17093–8. 10.1073/pnas.1114194108 PMC3193242 21949249

[54] Nakamura T Furukawa A Uchida K Ogawa T Tamura T Sakonishi D Autophagy Induced by Intracellular Infection of Propionibacterium acnes. PloS One (2016) 11:e0156298. 10.1371/journal.pone.0156298 27219015 PMC4878785

