# Supplementary figures and images for: Cutibacterium acnes Infection Induces Type I Interferon Synthesis Through the cGAS-STING Pathway
Source: Front Immunol. 2020 Oct 15;11:571334. doi: 10.3389/fimmu.2020.571334 (PMC7593769; doi:10.3389/fimmu.2020.571334)

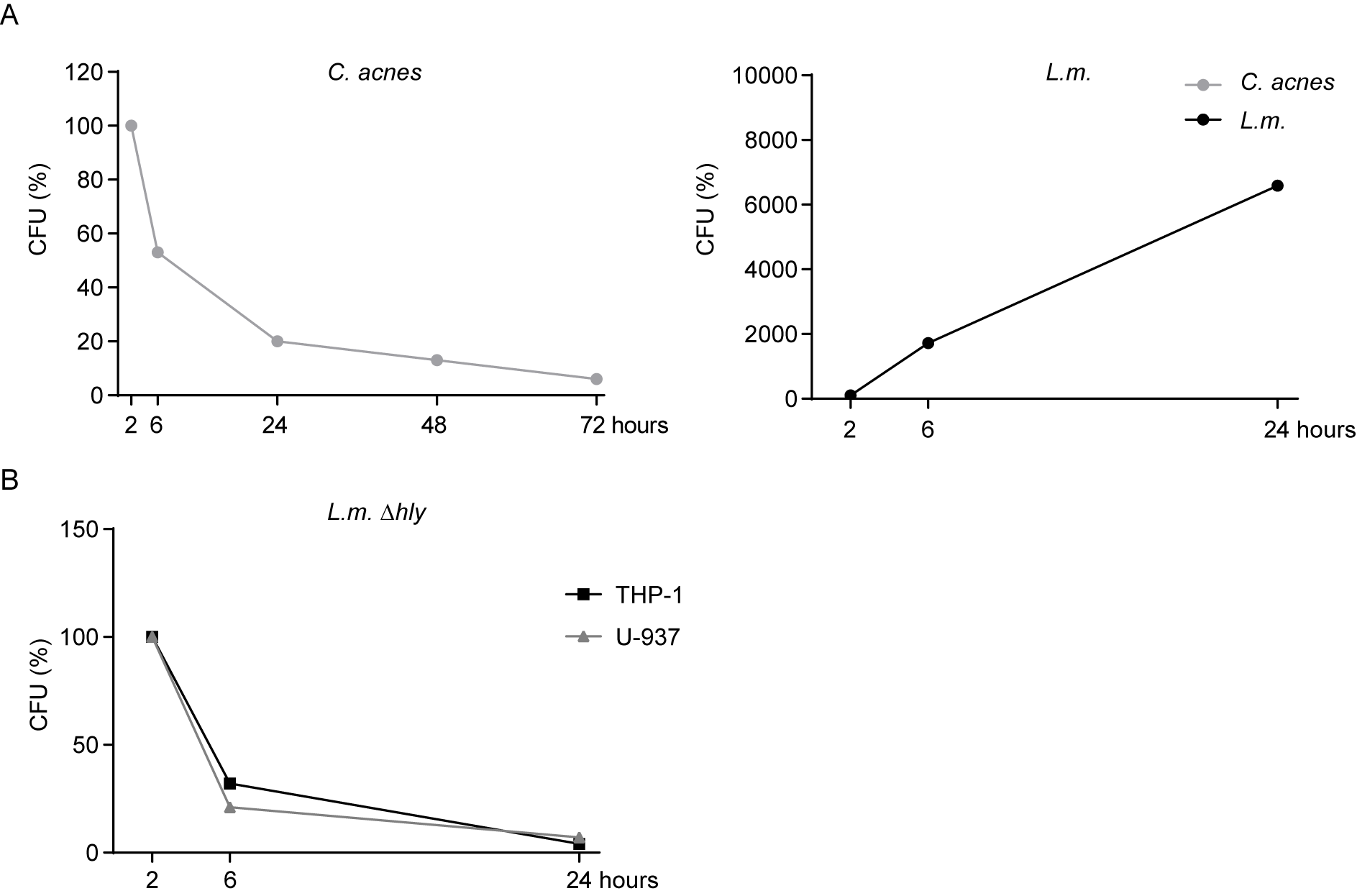

Supplement: Supplementary Figure 1 — (A) CFU assay was performed using PMA-differentiated U-937 cells. U-937 cells were infected with either C. acnes strain NCTC737 (left; grey) or wildtype L.m. (right; black) for indicated timepoints. (B) CFU assay was performed using PMA-differentiated THP-1 (black squares) and U-937 (grey triangles) cells. Cells were infected with the L.m. mutant strain Δhly for indicated timepoints. (A–B) Representative of two independent experiments. [file Image_1.tif]

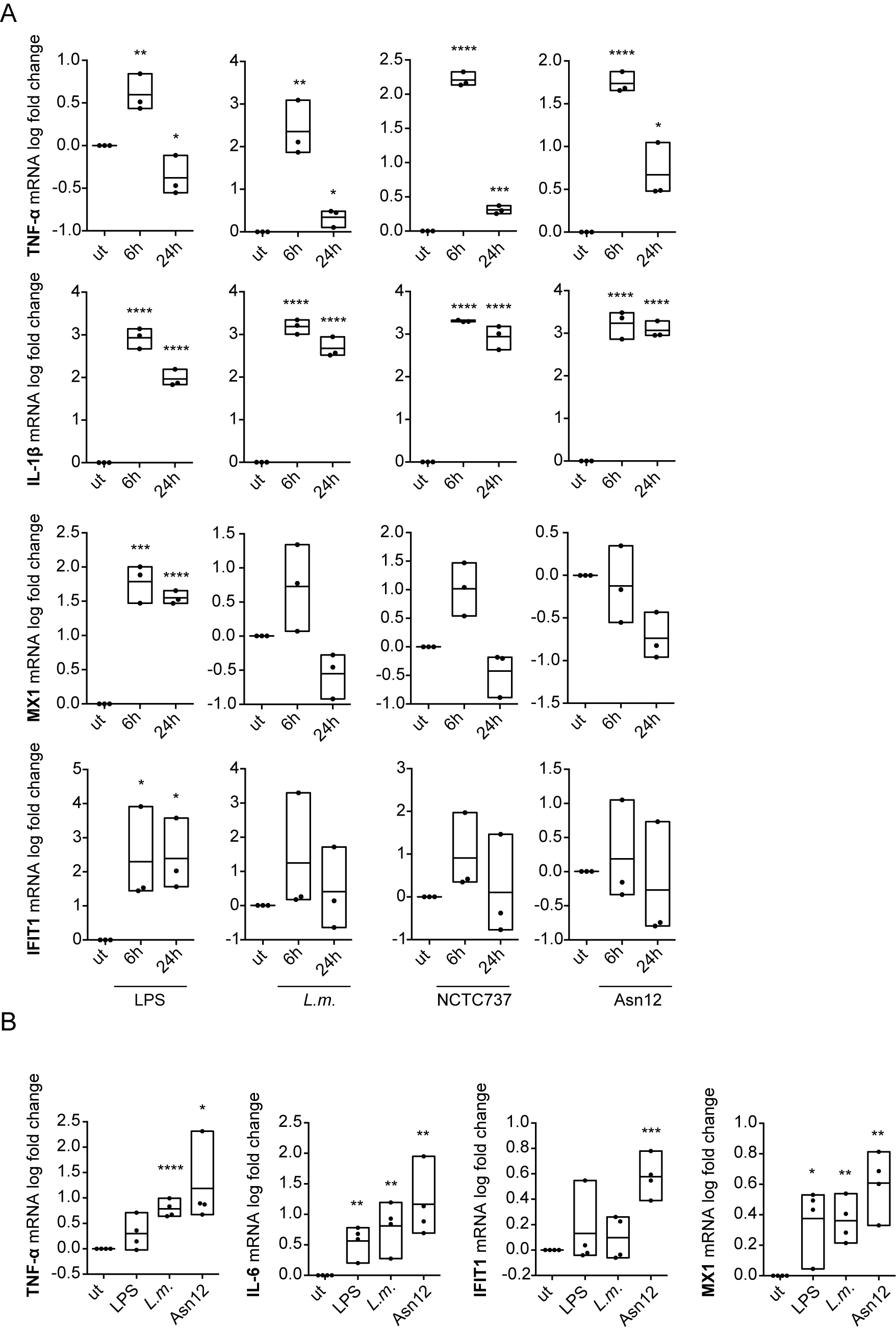

Supplement: Supplementary Figure 2 — (A) Primary monocytes were infected with either C. acnes strain NCTC737 and Asn12, L.m. or stimulated with LPS for either 6 or 24 h. HPRT-normalized gene expression of TNF-α, IL-1β, MX1 and IFIT1 was measured using RT-qPCR and shown as log transformed fold change to the uninfected sample. (B) HaCaT cells were infected with either C. acnes strain Asn12, L.m. or stimulated with LPS for 48 h. HPRT-normalized gene expression of TNF-α, MX1, IFIT1 and IL-6 was measured using RT-qPCR and shown as log transformed fold change to the uninfected sample. (A–B) Data represent the mean values of three independent experiments. P. values were calculated using the unpaired t-test of log transformed values (*P ≤ 0.05; **P ≤ 0.01; ***P ≤ 0.001). [file Image_2.tif]

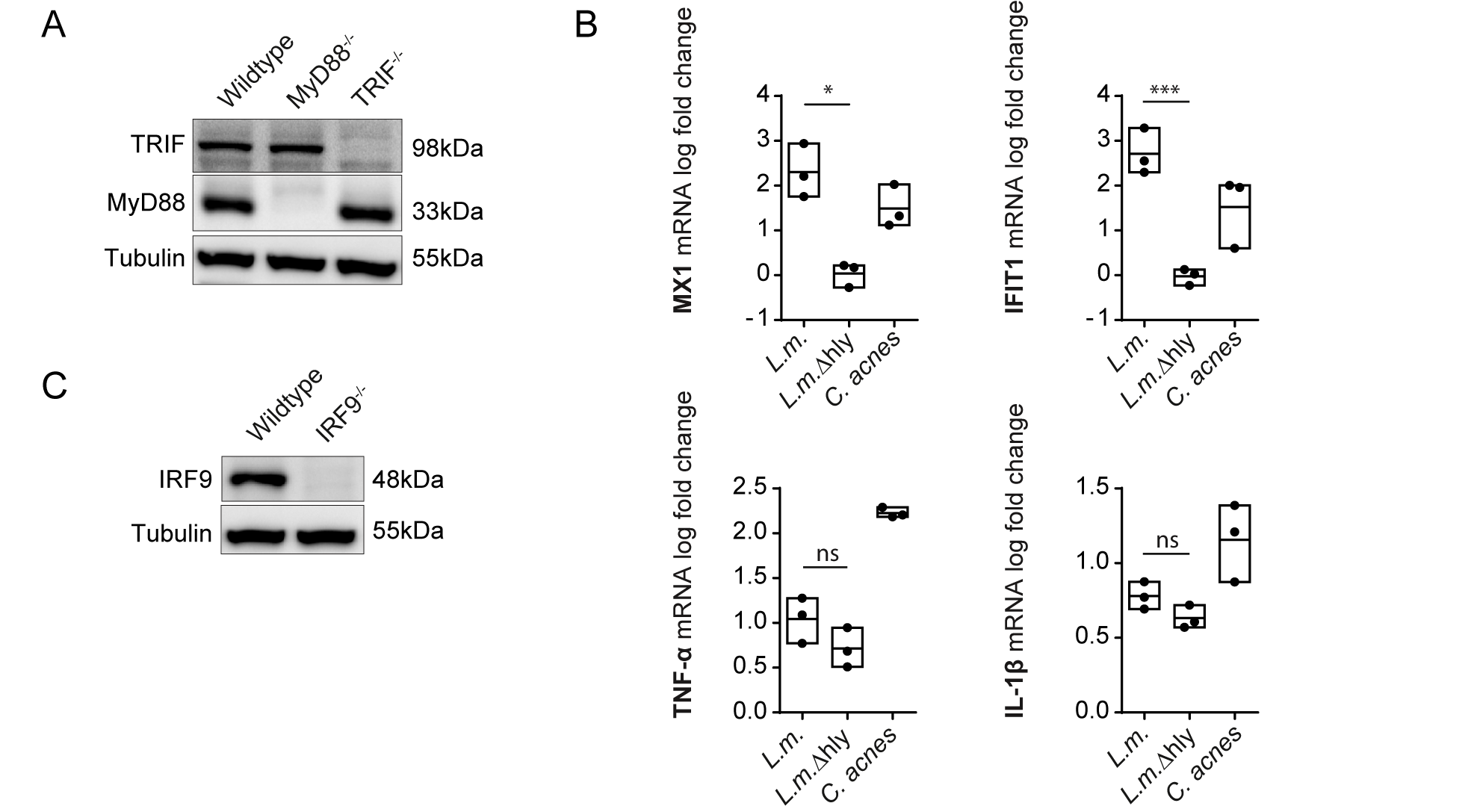

Supplement: Supplementary Figure 3 — (A) Confirmation of CRISPR/Cas9 generated MyD88 and TRIF knockout THP-1 cells. MyD88, TRIF and tubulin protein levels of wildtype, MyD88 and TRIF knockout THP-1 cells was analyzed by western blot. (B) Differentiated THP-1 cells were infected with either C. acnes strain NCTC737, wildtype L.m. or LLO-deficient L.m. strain Δhly for either 6 (TNF-α, IL-1β) or 24 h (MX1, IFIT1). HPRT-normalized gene expression was measured using RT-qPCR and shown as log transformed fold change to the uninfected sample. Data represent the mean values of three independent experiments. P. values were calculated using the unpaired t-test of log transformed values (*P ≤ 0.05; **P ≤ 0.01; P***≤0.001). (C) Confirmation of CRISPR/Cas9 generated IRF9 knockout THP-1 cells. IRF9 protein level of wildtype and IRF9 knockout THP-1 cells was analyzed by western blot. [file Image_3.tif]

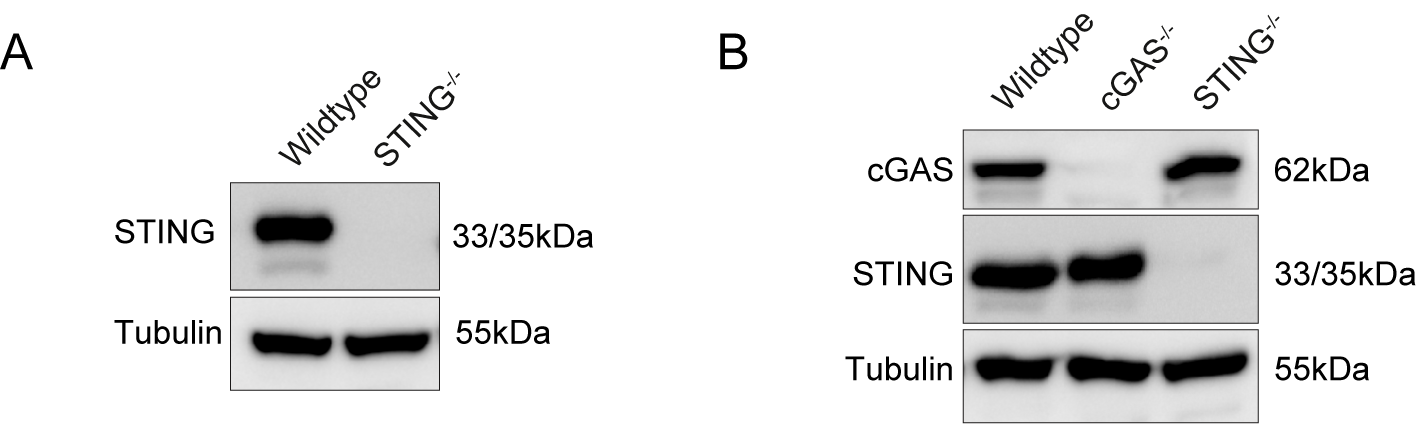

Supplement: Supplementary Figure 4 — (A) Confirmation of STING knockout THP-1 cells. STING and tubulin protein levels of wildtype and STING knockout THP-1 cells was analyzed by western blot. (B) Confirmation of cGAS knockout THP-1 cells. STING, cGAS and tubulin protein levels of wildtype, cGAS and STING knockout THP-1 cells was analyzed by western blot. [file Image_4.tif]
