# Supplementary material for: Cutibacterium acnes Infection Induces Type I Interferon Synthesis Through the cGAS-STING Pathway
Source: Front Immunol. 2020 Oct 15;11:571334. doi: 10.3389/fimmu.2020.571334 (PMC7593769; doi:10.3389/fimmu.2020.571334)
Supplement: Supplementary file 5 [file Table_1.docx]

| **Strain** | **Phylotype** | **Subspecies** | **MLST_8_ data*^a^*** | | **Source** | **WGS accession** |
| --- | --- | --- | --- | --- | --- | --- |
|  |  |  | **ST** | **CC** |  |  |
| NCTC737*^b^* | IA_1_ | *acnes* | 1 | 1 | Acne | NZ_CP023676 |
| P.acn31 | IA_2_ | *acnes* | 36 | 2 | Aqueous humour | NC_016511 |
| KPA171202 | IB | *acnes* | 5 | 5 | Contaminant | NC_006085 |
| PV66 | IC | *acnes* | 85 | 107 | Acne | - |
| ATCC11828*^b^* | II | *defendens* | 27 | Singleton | Abscess | NC_017550 |
| Asn12 | III | *enlongatum* | 33 | 77 | Prosthetic hip | NZ_QKRC01000001 |

**Table S1: Molecular and clinical information on the strains used in this study.**

*^a^*ST= sequence type; CC= clonal complex (https://pubmlst.org/cacnes/)

*^b^*Type strains
